# Comparison of antibiotic resistance genes in swine manure storage pits of Iowa, USA

**DOI:** 10.3389/frabi.2023.1116785

**Published:** 2023-03-23

**Authors:** Timothy P. Neher, Michelle L. Soupir, Daniel S. Andersen, Maggie L. O’Neill, Adina Howe

**Affiliations:** Department of Agricultural and Biosystems Engineering, Iowa State University, Ames, IA, United States

**Keywords:** antimicrobial resistance, livestock management, production system, integrator, manure storage, swine manure, qPCR (quantitative PCR), high-throughput qPCR

## Abstract

Antimicrobial resistance (AMR) can develop in deep-pit swine manure storage when bacteria are selectively pressured by unmetabolized antibiotics. Subsequent manure application on row crops is then a source of AMR into soil and downstream runoff water. Therefore, understanding the patterns of diverse antibiotic resistance genes (ARGs) in manure among different farms is important for both interpreting the results of the detection of these genes from previous studies and for the use of these genes as bioindicators of manure borne antibiotic resistance in the environment. Previous studies of manure-associated ARGs are based on limited samples of manures. To better understand the distribution of ARGs between manures, we characterized manures from 48 geographically independent swine farms across Iowa. The objectives of this study were to characterize the distribution of ARGs among these manures and to evaluate what factors in manure management may influence the presence of ARGs in manures. Our analysis included quantification of two commonly found ARGs in swine manure, *ermB* and *tetM*. Additionally, we characterized a broader suite of 31 ARGs which allowed for simultaneous assays of the presence or absence of multiple genes. We found the company integrator had a significant effect on both *ermB* (*P=0.0007*) and *tetM* gene concentrations (*P=0.0425*). Our broad analysis on ARG profiles found that the *tet(36)* gene was broadly present in swine manures, followed by the detection of *tetT*, *tetM*, *erm(35)*, *ermF*, *ermB*, *str*, *aadD*, and *intl3* in samples from 14 farms. Finally, we provide a comparison of methods to detect ARGs in manures, specifically comparing conventional and high-throughput qPCR and discuss their role in ARG environmental monitoring efforts. Results of this study provide insight into commonalities of ARG presence in manure holding pits and provide supporting evidence that company integrator decisions may impact ARG concentrations.

## Introduction

Large-scale swine production and growing demand for pork has resulted in the consequent increased production of swine manures (OECD and Food and Agriculture Organization of the United Nations, 2021). Manures are a reservoir for unmetabolized antibiotics and antibiotic resistant bacteria (Marti et al., 2014; Mu et al., 2015; He et al., 2020; Lima et al., 2020; Howe and Soupir, 2021). The enrichment of antibiotics in manure originates from the use of antibiotic administration to therapeutically and sub-therapeutically control, prevent, and treat disease (Klein et al., 2018). In the United States, more than two million kilograms, or 39% of medically important antibiotics intended for use in food-producing animals, were used in swine production in 2019 (Center for Veterinary Medicine, 2020). Much of the administered antibiotic is unmetabolized and remains in the animal tissue or excreted with manure (Elmund et al., 1971; Bacanlı and Başaran, 2019). Excess manure and associated antibiotic residues are often retained in deep pit storage structures until field application as fertilizer (Elmund et al., 1971; Zhang et al., 2017). Manure can remain in storage structures for more than a year, between intervals of land application (IADNR, 2022). Within these deep pits, there is continuous interaction between antibiotics and bacteria, which can lead to the development and/or enrichment of antibiotic resistance, both by genetic mutation and horizontal gene transfer (Chee-Sanford et al., 2009; Zhao et al., 2019; He et al., 2020). Generally, manure has been identified as a potential hotspot for the accumulation and dissemination of antibiotic resistance to the environment.

Diverse antibiotic resistant genes (ARGs) associated with medically important classes of antibiotics have been observed in swine manure bacteria. Swine manure associated ARGs include tetracyclines (*tet*), macrolides (*erm*, *msr*, *mef*), lincosamides (*lnu*, *lin*), aminoglycosides (*aac*, *aad*, *aph*, *str*), sulfonamides (*sul1*, *sul2*), amphenicols (*cpr*, *cml*, *floR*), and fluoroquinolones (*qnr*), ranked by total mass distributed in the US. (Fang et al., 2018; Center for Veterinary Medicine, 2020; Checcucci et al., 2020). The most commonly detected ARG determinants in swine manure encode resistance to tetracyclines (*tet*), sulfonamides (*sul*), and macrolides (*erm*) (Chen et al., 2007; Whitehead and Cotta, 2013; Li et al., 2019). A number of these ARGs have been detected within environments adjacent to animal production or manure application and are attributed to manure management practices (Wang et al., 2020), supporting the theory that manure-borne antibiotics and subsequent antimicrobial resistance contribute to the overall resistome in environmental soil and water (Wellington et al., 2013; Checcucci et al., 2020; Zhou et al., 2020).

To understand the risk of AMR from swine manure, broad and effective surveillance methods are necessary. Ideally, these methods would be sensitive and specific to swine-specific AMR risks, such as ARGs or pathogens. Unfortunately, the ARGs that are associated with swine manures are also detected in other animal production where similar antibiotics are used (Zalewska et al., 2021). Furthermore, ARGs and antibiotic resistant bacteria are naturally occurring in the environment (Martínez, 2012; Van Goethem et al., 2018), making it necessary to distinguish antibiotic resistance determinants derived from swine production to those that are found in the natural environment (Allen et al., 2010; Meyers et al., 2020). Additionally, swine manures themselves can vary significantly in the suite of ARGs that are characteristic of their microbial communities (Xue et al., 2021; Shui et al., 2022). We have a limited understanding of this variation among manures because most studies of swine-associated ARGs have been focused on demonstrating an enrichment of ARGs in a small sample of a single farm or a small number of manure samples (Li et al., 2019; Wen et al., 2019; Yang et al., 2020; Xue et al., 2021).

We focused on swine manures originating from the state of Iowa, which is the highest swine producing state in the United States, where there are more than 5,400 swine farms (IPPA, 2012). The rationale for selecting a state-wide sampling was based on accessibility to samples within a similar time period and also our expectation that we would observe high variability in swine production systems and company integrators within regional samples. Swine farms can vary in specialized production systems such as wean-finish or grow-finish, and company integrators that manage supplies like weaners, feed, and medication (Cooper, 2018). It is yet unclear how these variables may influence resulting AMR in stored manure.

In this study, we expand our knowledge of the presence of antibiotic resistant determinants in swine manure by providing a broad comparison of ARGs among manures from 48 farms. We aimed to quantify the presence of ARGs that have been demonstrated to be consistently enriched in swine manures, *tetM* and *ermB* (Whitehead and Cotta, 2013; Wen et al., 2019; Alt et al., 2021) and also characterized the presence of diverse resistance genes associated with other antibiotics and with swine manure, including aminoglycoside, carbapenem, lincosamide, phenicol, and sulfonamide resistance (**Table 1**). Our justification for the gene selection is that these genes are associated with the most sold antibiotics in swine production (Center for Veterinary Medicine, 2020). Additionally, a parallel study of the manures from these farms measured high levels of tetracyclines and macrolides (Congilosi et al., 2022). Our objective of this study was to better understand ARG representation across multiple swine sources in a similar region and to assess the variability of ARGs in swine manure and their usefulness as broad bioindicators of manure influence. Concurrently with evaluating ARGs among farm manures, we assessed the differences in farm management: production system (wean-finish or grow-finish) and company integrator (integrator 1 or integrator 2). Understanding the distribution of these genes under varying farm management conditions will help us better understand whether broad management factors influence the concentrations of manure-associated ARGs in swine manure from deep pit storage structures.

**Table 1:** Antibiotic resistance genes observed in previous studies in soil and water influenced by swine manure.

Antibiotic class of resistance	Antibiotic Resistance Gene	Studies reported
Aminoglycoside	*aadD, aada2, str*	(Chen et al., 2017; Han et al., 2018; Liu et al., 2019)
Carbapenem	*blaPSE, blaOXA10*	(Han et al., 2018; Cheng et al., 2020; Radu et al., 2021)
Lincosamide	*lnuA, lnuB*	(Han et al., 2018; Cheng et al., 2020)
Macrolide	*erm(35), erm(36), ermB, ermC, ermF, ermQ, ermT*	(Chen et al., 2017; Peng et al., 2017; Han et al., 2018; Liu et al., 2019; Lopatto et al., 2019; Wen et al., 2019; Meyers et al., 2020; Radu et al., 2021)
Mobile Genetic Element	*intI1, intI2, intI3, intI1F165*	(Chen et al., 2019; Lopatto et al., 2019; Meyers et al., 2020)
Phenicol	*floR, cmlA1, cmlA5*	(Chen et al., 2017; Liu et al., 2019)
Sulfonamide	*sul1, sul2*	(Peng et al., 2017; Chen et al., 2019; Liu et al., 2019; Lopatto et al., 2019; Meyers et al., 2020; Radu et al., 2021)
Tetracycline	*tet(36), tetA, tetL, tetM, tetO, tetT, tetW, tetX*	(Chen et al., 2017; Chen et al., 2019; Peng et al., 2017; Han et al., 2018; Liu et al., 2019; Wen et al., 2019; Meyers et al., 2020; Radu et al., 2021)

## Materials and methods

### Sample collection

A total of 48 swine farms were sampled from across the state of Iowa in the summer of 2020. At each farm, a single representative manure sample was collected from deep pit storage structures. Specific locations of the farms are not disclosed due to privacy restrictions, but all farms are within the state of Iowa and are geographically independent of each other. Samples were collected at the edge of the pits through a manure pump out *via* dipping the sample from the top six inches of the manure surface. All farms were deep pit barn facilities where pigs were either grow-finish (GF) or wean-finish (WF) pigs raised on a slatted floor. Pigs were fed commercial production diets consisting primarily of corn, soybean meal, and distillers grains with percentages fed varying by growth stage and price of different feed ingredients. After collection, manure was stored at -20°C for one month until further processing. Each manure sample was subsampled in triplicate prior to DNA extraction. Each farm included was categorized based on originating integrator and production system. Specifically, these categories were company integrator: Integrator 1 (n=24) or integrator 2 (n=24); production system: wean-finish (n=34), or grow-finish (n=14). Ethical review and approval was not required for the study on animals in accordance with the local legislation and institutional requirements. This work was conducted in collaboration with local swine growers who made all animal decisions regarding health and well-being and allowed the collection of manure at their site.

### DNA extraction

The DNA extraction procedure followed protocols from the MagAttract PowerSoil DNA EP Kit (Qiagen) and an epMotion 5075 automated robot for extraction (Eppendorf). Samples of 0.25 grams wet weight of liquid swine manure were used for DNA extraction. Each manure was sub-sampled into three replicates (“farm replicates”). For each farm replicate, we performed three DNA extractions, resulting in three technical extraction replicates (“extraction technical replicates”) for each farm replicate manure sample. The resulting DNA was cleaned using a DNA Clean and Concentrator kit (Zymo Research). Subsequent DNA concentrations were measured with the Quant-it dsDNA Assay Kit, high sensitivity (Thermo Fisher Scientific). The DNA samples were stored at -80°C until further use.

### Conventional qPCR quantification (quantification of concentrations of *tetM* and *ermB*)

Conventional qPCR assays were performed on a CFX96 Touch Real-Time PCR Detection System (BioRad) and measured in triplicate using primers targeting the 16S rRNA gene, *ermB* gene, and *tetM* gene (**Supplementary Table 1**). Genes were quantified in all 48 swine manure samples. The DNA template was diluted (1:10) to optimize qPCR detection, to minimize inhibitors, and increase primer efficiency to a target gene. The limit of quantification was determined for each gene using oligonucleotide standards. Standard curves ranged from 10^7^ to 10^1^ copies, and all samples measured above the limit of quantification. Outliers in the triplicate were omitted if above 1.5 times the standard deviation in the average of the three values. Efficiencies calculated by standard curves ranged from 82.2 to 100.6% and all R^2^ values were above 0.98 (**Supplementary Table 2**). All reported absolute abundance (copies/gram) are reported in gene copies per gram of wet weight of manure and were calculated by the equation:


$$=\frac{x\;copies}{reaction}*\frac{100\mu L\;final\;volume}{\frac{2\mu L}{reaction}}*dilution\;factor\;10*\left(\frac{1}{0.25g\;manure}\right)$$

### High-throughput qPCR (presence absence of ARGs)

Extracted DNA was analyzed for a wide host of ARGs encoding resistance to a broad spectrum of antibiotics used in swine production; *str*, *aadD*, *aadA2* (Aminoglycosides), *ermB*, *ermC*, *ermF*, *ermQ*, *ermT*, *erm(35)*, *erm(36)* (Macrolides), *sul2*, *sul1* (Sulfonamides), *tetA*, *tetL*, *tetM*, *tetO*, *tetT*, *tetW*, *tetX*, *tet(36)* (Tetracyclines), *blaPSE*, *blaOXA10* (Carbapenems), *lnuC*, *lnuA* (Lincosamides), *cmlA5*, *cmlA1*, *floR* (Phenicols), *intI3*, *intI2*, *intI1F165*, and *intI1* (Integrons). The high-throughput qPCR primers used for the analysis are originally described in Stedtfeld et al. (2018), **Supplementary Table 1**. The high-throughput qPCR assay was performed on the Biomark Fluorescent machine in the 96x96 primer target layout. Each assay was performed in triplicate. The template DNA was diluted in a 1:500 dilution for optimal performance on the Biomark machine and to decrease potential inhibitor effects. Samples reading a cycle threshold value greater than 30 were omitted from further analysis. Cycle threshold detections greater than 30 were assumed to be non-detected. Verification of the high-throughput qPCR machine performance is supported with internal standards for standard curve development of 16S rRNA, *ermB*, *ermF*, *sul2*, *tetM*, and *tetW* genes. Each internal standard gene amplified successfully with efficiencies ranging from 80.0 to 104.2% (**Supplementary Table 3**).

### Quality control

In order to be deemed a successful amplification, we required that the conserved total bacteria gene 16S rRNA was detected in each manure sample. Additionally, we required that detection was observed for each gene in 2 out of 3 farm manure replicates and 2 out of 3 technical extraction replicate detections for each sample.

### Statistical analysis

All statistical analyses were performed using R version 4.0.3. The quantified *ermB* and *tetM* gene concentrations (copies/gram wet weight) were log10 transformed to fit a normal distribution. Normality was confirmed with visual inspection of histograms and Q-Q Plots. The linear regression models were fit using the lme4 package (Bates et al., 2015). The two gene responses were analyzed separately. The integrator and production system were treated as fixed effects. Gene concentrations of subsampled triplicates from one representative manure sample per farm were averaged before model building. Model performance was evaluated using the Performance package (Lüdecke et al., 2021) (**Supplementary Table 4**).

The R package emmeans (Lenth, 2021) was used for calculating the estimated marginal means from the verified models and making pairwise comparisons of fixed effects. All pairwise comparisons were made with a 95% confidence level (*P<0.05*) and P-values were adjusted using Tukey’s method for multiple comparisons. The main effects refer to the overall effect of the variable while ignoring, or averaging over, the levels of the other predictor variable. The main and interaction effects of each model were analyzed using ANOVA and type-III error.

## Results

### Conventional qPCR gene quantification

**Figure 1:**
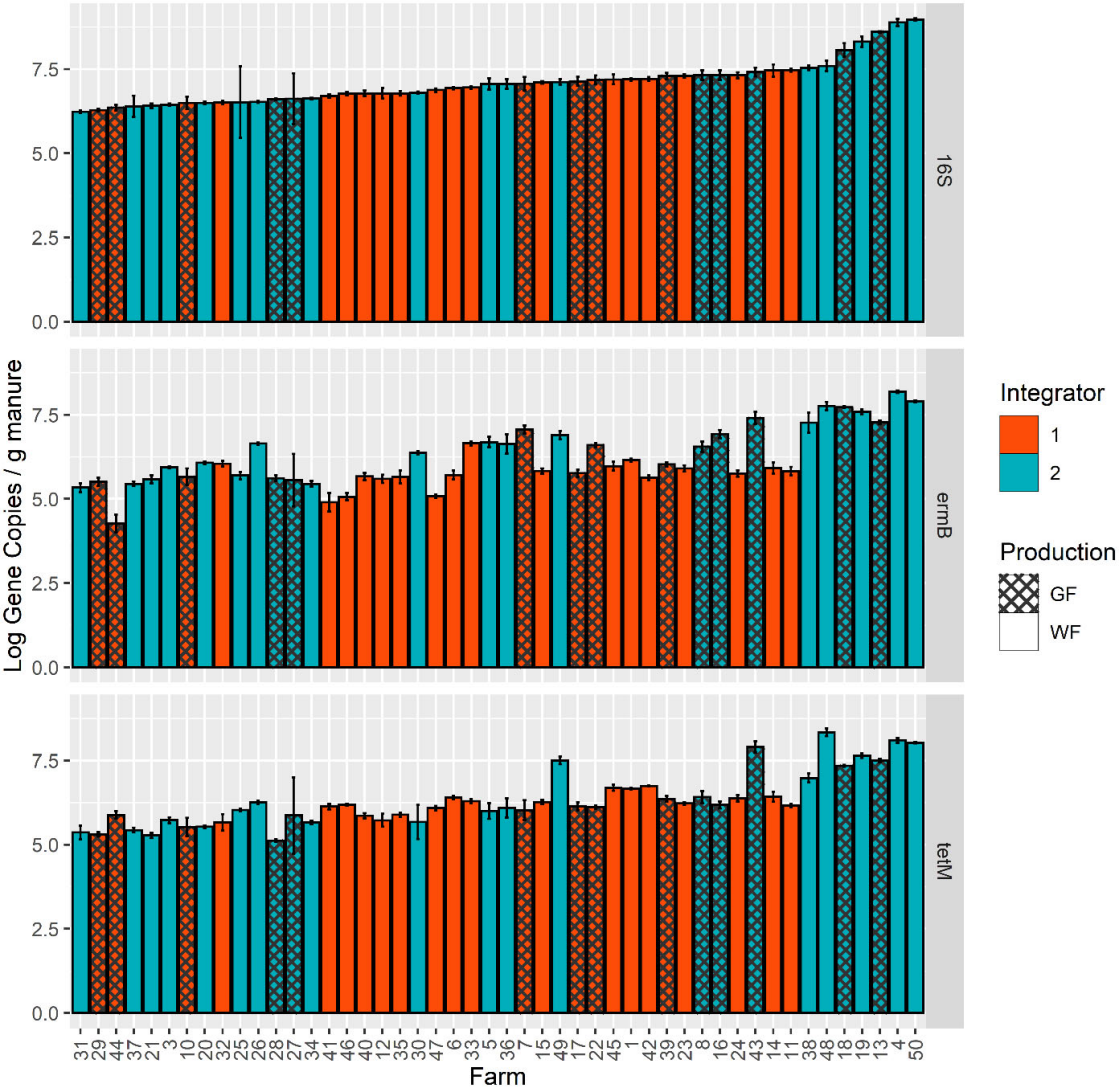
Absolute gene copies/g (wet weight) of 16S rRNA, *ermB*, and *tetM* as measured by qPCR assays for 48 farm manure samples. Samples are ordered by lowest to highest mean concentrations for the 16S rRNA gene. Colors indicate the different company integrators, and the hash marks denote the growth stage (production of the farm, GF (Grow-Finish) and WF (Wean-Finish)).

The number of gene copies of *tetM* and *ermB* were quantified in DNA extracted from all manures using targeted amplification of these genes. Additionally, gene copies of the 16S rRNA gene, a phylogenetic marker present in all bacteria, were estimated and used for normalizing total bacterial counts among manure comparisons. Overall, there was a large range of detection of both genes across all 48 farms (**Figure 1**); the absolute gene concentrations of *ermB* ranged from 2.20x10^4^ copies gram^-1^ to 1.53x10^8^ copies gram^-1^ and *tetM* ranged from 1.33x10^5^ copies gram^-1^ to 2.23x10^8^ copies gram^-1^. The limit of quantification for each individual qPCR plate are reported in **Supplementary Table 2**. The concentrations of *ermB* and *tetM* were significantly different across the 48 manure samples (ANOVA, *P< 0.0001*) (**Supplementary Table 5**), and a general trend was observed that *tetM* and *ermB* concentrations increased with concentrations of 16S rRNA genes.

**Figure 2:**
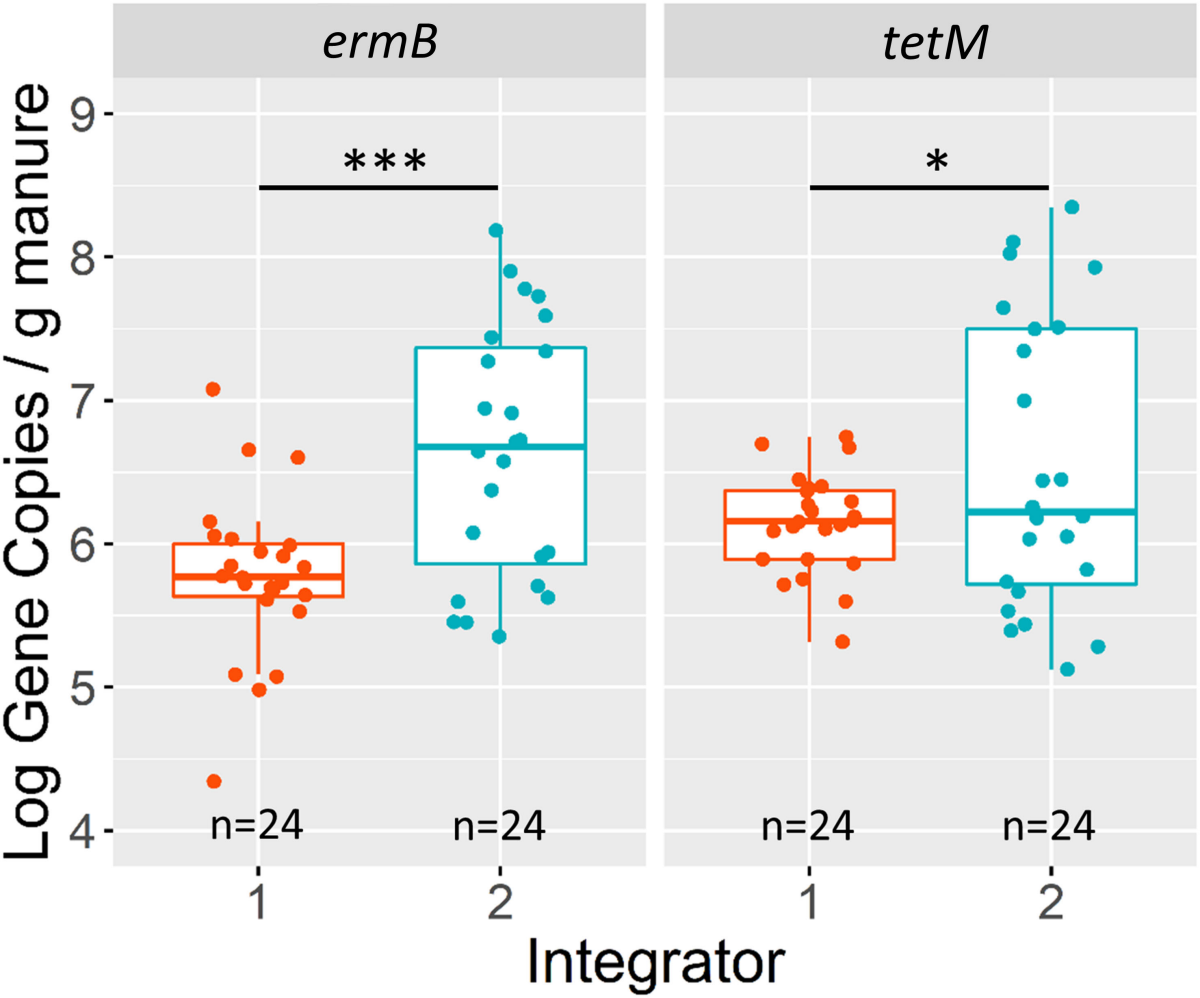
Log10 gene copies/g (wet weight) of *ermB* and *tetM* grouped by company integrator. Asterisks above boxplots signify p-values (alpha = 0.05) based on results of the linear model (not significant [ns] p>0.05, *p<0.05, **p<0.01, ***p<0.001, ****p<0.00001). Interquartile ranges are indicated by boxes and the upper 25% and lower 25% are indicated by whiskers. The number of farms (n) are labelled on the x-axis.

The company integrator had a significant main effect on observed *ermB* absolute gene concentrations based on the overall ANOVA with type-III error (*P=0.0007*) (**Supplementary Table 6**). The mean concentration of *ermB* in manures associated with integrator 2 manure was 15% greater than manures from integrator 1. Integrator 2 had an *ermB* estimated marginal mean of 4.8x10^6^ copies/gram compared to integrator 1 with 6.5x10^5^ copies/gram. This result exists when *ermB* was normalized to 16S rRNA (*P=0.0020*) (**Supplementary Figure 1** and **Supplementary Table 7**). Likewise, there is evidence that the integrator had a significant effect on *tetM* concentrations (*P=0.0425*), with *tetM* also being enriched in integrator 2 relative to integrator 1 (**Figure 2**). However, this result is non-significant when *tetM* was normalized to 16S rRNA (*P= 0.3670*). The production system had no significant main effect on *ermB* or *tetM* gene concentrations or relative abundance to 16S rRNA (**Supplementary Figures 2, 3**). Additionally, there was no significant interaction between the two fixed effects in both the absolute copy number model and the 16S rRNA normalized model for each gene (**Supplementary Tables 6, 7**).

### HT-qPCR gene survey

**Figure 3:**
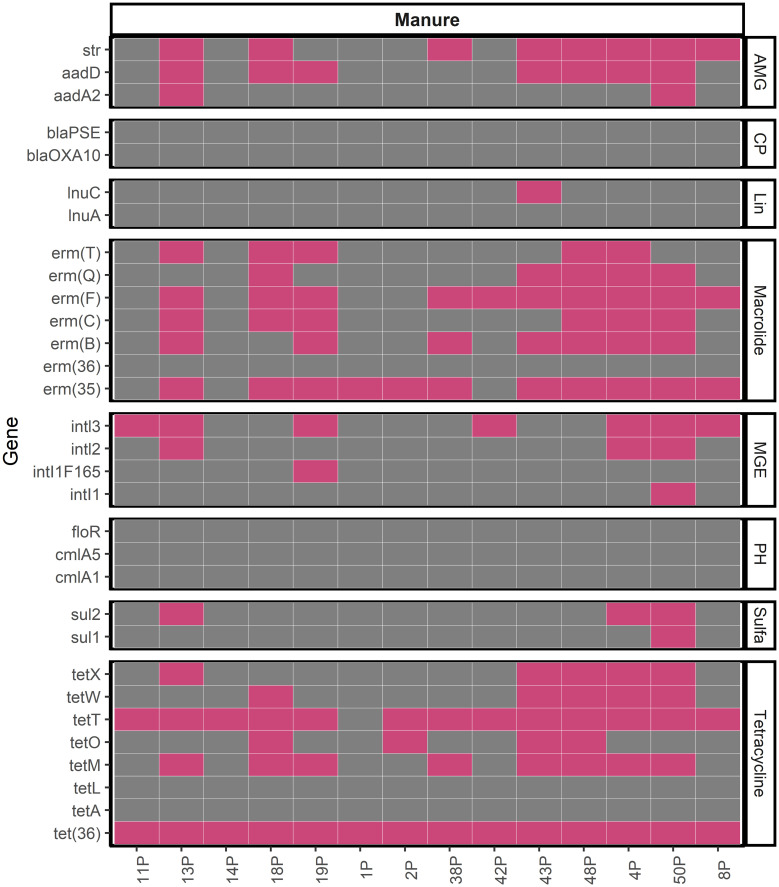
Presence (Pink) and absence (Grey) for ARGs in manure samples for which amplification of 16S rRNA gene was observed. Aminoglycoside (AMG), Carbapenem (CP), Lincosamide (Lin), Macrolide, Mobile Genetic Element (MGE), Phenicol (PH), Sulfonamide (Sulfa), Tetracycline.

In addition to quantification of *tetM* and *ermB* in manures, we also evaluated the presence of 31 ARGs listed in **Table 1** and the 16S rRNA gene in manures using methods similar to those previously described (Stedtfeld et al., 2018) to leverage the ability to assay numerous genes simultaneously with high-throughput qPCR (HT-qPCR). Each internal standard gene of 16S rRNA, *ermB*, *ermF*, *sul2*, *tetM*, and *tetW* were amplified successfully with efficiencies ranging from 80-104% (**Supplementary Table 3**). However, while all 48 manures were evaluated against these 32 genes, in total, we detected 22 unique ARGs in 14 independent farm manure samples (**Figure 3**). In 34 manures, we were unable to amplify the 16S rRNA gene with HT-qPCR assays and thus these samples were removed from further analysis. Within successfully amplified samples, the most frequently detected ARG in manure was *tet(36)*, which was detected in all 14 manures. The second most detected ARG was *tetT* at 93% detection, followed by *erm(35)* at 78.6% detection. Genes encoding resistance to tetracycline, *tetT*, *tetM*, and *tet(36)*, were present in 13/14, 8/14, and 14/14 farm manure samples, respectively. The macrolide resistance gene class, *erm*, had the second most detected antibiotic resistance genes with *erm(35)*, *ermF* and, *ermB* detected in 11/14, 10/14, and 7/14, respectively. There was no detection of *blaPSE*, *blaOXA10*, *cmlA5*, *cmlA1*, *floR*, *lnuA*, *erm(36)*, *tetL*, and *tetA* in any of the manure samples.

Based on the detection of ARGs, we have developed recommendations of the most commonly detected ARGs in Iowa swine manures (**Table 2**). Importantly, we also identify the ARGs that were not strongly present in manure holding pits, and these ARGs include *tetL*, *tetA*, *erm(36)*, *floR*, *cmlA5*, *cmlA1*, *blaPSE*, and *blaOXA10* (no detection), *sul1*, *inti1*, *and inti1F165*, (7.1%), *aadA2* (14.2%), *inti2* and *sul2* (21.4%). In general, we observed that the two main resistance mechanisms of ARGs present in the manures studied were associated with target protection and target alteration.

**Table 2:** Ranked recommendations of ARGs for detection of AMR in swine manure holding pits, based on both detection of 16S rRNA genes and specified ARG.

Gene	Percent Detection	Drug Class	Resistance Mechanism
*tet(36)*	100	Tetracycline	Target protection
*tetT*	92.9	Tetracycline	Target protection
*erm(35)*	78.6	Macrolide	Target alteration
*ermF*	71.4	Macrolide	Target alteration
*tetM*	57.1	Tetracycline	Target protection
*str*	57.1	Aminoglycoside	Inactivation
*ermB*	50	Macrolide	Target alteration
*aadD*	50	Aminoglycoside	Inactivation
*intl3*	50	Integrase	N/A
*ermC*	42.9	Macrolide	Target alteration
*ermQ*	35.7	Macrolide	Target alteration
*ermT*	35.7	Macrolide	Target alteration
*tetW*	35.7	Tetracycline	Target protection
*tetX*	35.7	Tetracycline	Inactivation
*tetO*	28.6	Tetracycline	Target protection
*sul2*	21.4	Sulfonamide	Target replacement
*intl2*	21.4	Integrase	N/A
*aadA2*	14.2	Aminoglycoside	Inactivation
*intI1F165*	7.1	Integrase	N/A
*intI1*	7.1	Integrase	N/A
*sul1*	7.1	Sulfonamide	Target replacement
*lnuC*	7.1	Lincosamide	Inactivation
*lnuA*	1.6	Lincosamide	Inactivation
*tetL*	0	Tetracycline	Efflux
*tetA*	0	Tetracycline	Efflux
*erm(36)*	0	Macrolide	Target alteration
floR	0	Phenicol	Efflux
cmlA5	0	Phenicol	Efflux
cmlA1	0	Phenicol	Efflux
blaPSE	0	Carbapenem	Inactivation
blaOXA10	0	Carbapenem	Inactivation

The percent detection is the proportion of 14 manure samples with concurrent positive detection of 16S rRNA gene. The antibiotic resistance genes analyzed in this study in 14 swine manures from Iowa farms. N/A, Not Applicable.

## Discussion

Many previous studies have characterized ARGs in swine manures (Whitehead and Cotta, 2013; Yang et al., 2020; Howe and Soupir, 2021) but are limited in the numbers of manure from different farms represented in a single study. To help understand the broad presence of ARGs in swine manures, this study identified patterns in diverse manures from 48 geographically independent farms. These farms represented variations in company integrator and production system, thus providing an opportunity to assess generalized management factors. The ARGs selected for characterization in this study were based on previous research in environmental monitoring, and these genes have been previously detected in manure, manure amended soil, and in the downstream waters of agricultural land (Berendonk et al., 2015; Chen et al., 2019; Lima et al., 2020; Neher et al., 2020; Zhang et al., 2021). While we know these genes have been enriched in association with manures in experimental studies, observations of their abundances in environmental samples may not be able to be linked to a manure reference. In other words, in environmental monitoring, it is unknown if abundances observed of these genes are substantial. Understanding the distribution of these genes in manures will help us frame their observed abundances in the environment. While we acknowledge that a study of 48 regional farms is far from comprehensive, we believe that this study fills an important data gap on ARG bioindicators from broad manures within a single comparative study.

In our evaluation of ARGs as bioindicators for swine manure, we used two approaches on select genes. Our rationale for leveraging both these methods was to balance our abilities to accurately quantify relevant ARGs to understand the distribution of their presence in diverse manures while also providing a broad survey of multiple ARGs. The first method we used was conventional gene amplification with qPCR, which is an absolute quantification method using known standard concentrations to estimate specific gene concentrations within manures. To survey a broad range of genes, we also used a second method, which is a relative quantification method on a HT-qPCR platform. This method has recently been used by numerous studies (Muurinen et al., 2021; Fernanda et al., 2022; Flater et al., 2022; Kasuga et al., 2022; Mware et al., 2022; Samanta et al., 2022) because it allows for simultaneous presence/absence detection of numerous genes (Stedfeld). HT-qPCR is also limiting in the volume of each reaction (6.7 x 10^-3^ μL vs 2 μL in conventional qPCR), which directly influences its detection limits. Thus, these amplification methods, conventional qPCR and HT-qPCR, are complements, the former allowing for more sensitive quantification of a limited number of ARGs and the latter broad detection of numerous ARGs simultaneously. As with any amplification method for manure samples, both methods will be influenced and likely disproportionately by the sample complexity of manures, where inhibitors (which vary among manure samples) may prevent adequate amplification (Sidstedt et al., 2020; Waseem et al., 2020; Park et al., 2021). We provide a comparison of these methods to target ARGs in our swine manure samples below.

### Concentrations of *ermB* and *tetM* in swine manure pits (conventional qPCR)

Consistent with previous observations of the association and enrichment of *ermB* and *tetM* genes with manures (Whitehead and Cotta, 2013; Joy et al., 2014; Zalewska et al., 2021) and adjacent soils and waters (Peng et al., 2017; Zhang et al., 2021), we detected these genes in all 48 manures in this study. The concentrations measured in our study were consistent with those detected in manure holding pits measured in other studies (Mackie et al., 2006; Joy et al., 2013; Hall et al., 2020; Alt et al., 2021) and also demonstrate the wide variations of ARGs that can be observed within manures, with variations up to three-fold. The wide ranges of measured *ermB* and *tetM* in these manures may be caused by covariates in manure holding pits that have yet unknown implications on ARG concentrations after long-term exposure such as concentrations of heavy metals, manure pit additives, or changes in chemical properties such as pH or organic substrates (Hölzel et al., 2012; He et al., 2020). While it is clear that these ARGs are consistently observed between swine manures, it is less clear what the implications are of the magnitude and variability of these gene concentrations (*ermB* and *tetM* varying between 2.20x10^4^ and 1.53x10^8^ copies/gram in our samples). We speculate that the concentration of ARGs may be associated with the time spent in storage, with manure sampled right at defecation presumably containing different concentrations of ARGs than in manure stored for up to six months (Joy et al., 2014). Future studies of the relationship between these gene concentrations and to risks antibiotic resistance are much needed (Gullberg et al., 2011; Hughes and Andersson, 2017), and the results of this study provide some insight the variability of these concentrations in varying manures.

The abundances of these genes also followed observable patterns based on their farm of origin. We observed significant differences of *ermB* and *tetM* gene concentrations among farms with different company integrators, with both genes consistently largest in the same integrator. Integrators generally manage piglet source, feedstock, and veterinary practices (Tsoulouhas and Vukina, 1999; McBride and Key, 2003; Reimer, 2006). Our observations that different integrators have different concentrations of these genes suggest that these management decisions may affect ARG concentrations in manures (Lu et al., 2017; Ghanbari et al., 2019; Cheng et al., 2021). We did not observe any significant differences in *tetM* or *ermB* in association to the production system, or whether manure originated from wean or grow-finished pigs. This finding is consistent with previous studies who investigated the differences of ARGs in swine from the same farm over time and found that similar genes were consistently observed among samples from different stages in the production process (Petrin et al., 2019) and also at similar concentrations (Wen et al., 2019). Our results combined with these previous studies suggest that despite higher quantities of antibiotics administered to younger weanling pigs than mature growers (Dunlop et al., 1998; Dewey et al., 1999), the concentrations of these ARGs in manure do not change significantly. Overall, our results also indicate that the integrator is a larger source of variation among these genes than production stage and highlight the opportunity to engage in AMR stewardship towards integrators in partnership with farms (Hayes, 2022; Mitchell et al., 2022).

### Potential ARG indicators in swine manure pits (HT-qPCR)

We also studied the detection of other ARGs to expand this study beyond *ermB* and *tetM* by leveraging high-throughput qPCR (HT-qPCR) methods which allow simultaneous testing of multiple gene probes. ARG targets were selected based on published primers (Stedtfeld et al., 2018) of ARGs previously observed to be present in swine manures (**Table 1**). Between manures, the tetracycline resistance gene class was the most prevalently detected in our samples, which is consistent with its wide use in swine production (Center for Veterinary Medicine, 2020). Likewise, the macrolide resistance gene class, *erm*, had the second most detected antibiotic resistance genes and is consistent with previous literature (Whitehead and Cotta, 2013; Joy et al., 2014). For instance, a study by Wen et al. (2019) studied nine ARGs at 18 different swine farms and found *tetO* as the predominant gene in manure and *tetQ*, *tetW*, *ermB*, and *ermF* were identified as having the highest risk of spread to the soil and water environment through manure application. Moreover, a study by Mu et al. (2015) took manure samples right after defecation from swine in nine feedlots in China finding *oqxB* (plasmic mediated quinolone) as the highest detected ARG followed by *sul1*, *sul2*, *tetO*, *tetM*, and *ermB*. Surprisingly, *sul1* and *sul2* were only detected 11.1% and 23% respectively, in the manure storage pits from the current study, suggesting a temporal shift in ARG presence between fresh manure and stored manure. Finally, a study of manure from three swine farms in China measured 28 tetracycline resistance genes and reported detection of 22 with the most common genes *tetA*, *tetL*, *tetM*, and *tetG* (Zhu et al., 2013), whereas in the current study, *tetA* and *tetL* were not detected in any of the 14 farms. These variations in detected classes of ARGs among studies and farms are speculated to be caused by differing antibiotic treatments, legacy resistance in piglets passed down by the maternal gut (Pärnänen et al., 2018), and co-selection of resistance genes (Looft et al., 2012).

Compared to conventional qPCR, fewer detections of ARGs were observed on HT-qPCR, most likely due to a combination of both the significantly reduced reaction volume (and thus lower limit of quantification) and presence of inhibitors (Funes-Huacca et al., 2011; Sandberg et al., 2018; Luo et al., 2021; Keenum et al., 2022). Specifically, we observed *ermB* and *tetM* gene detection in 100% of manure samples with conventional qPCR but 50% with HT-qPCR. To better understand these results, we compared the lower limit of quantification for *ermB* and *tetM* for traditional qPCR and HT-qPCR and found that traditional qPCR was 63 (*ermB*) and 94 (*tetM*) times more sensitive than HT-qPCR (**Supplementary Tables 2, 3**), suggesting that limit of quantification contributed to the inconsistency among ARG detections. Additionally, the DNA for the HT-qPCR assays were diluted 500:1 to balance measuring high 16S-rRNA gene copies, enabling the detection of low concentration ARGs, and reducing inhibitor effects. We conclude that the combination of diluting DNA and the HT-qPCR’s significantly reduced reaction volume contributed to the inconsistent detection of ARGs. This observation should be considered in selecting monitoring methods for ARG detection in future studies. Although HT-qPCR is not as robust as conventional qPCR, the advantages of this method are its ability to simultaneously measure multiple gene targets, use of much less reagent per sample, and significantly reduced labor. We recommend that HT-qPCR be used to screen the presence or absence of diverse ARG targets in environmental samples, and conventional qPCR be used for more rigorous quantification.

While *ermB* and *tetM* were inconsistently detected with HT-qPCR methods, there were specific genes that were broadly present using this method. Specifically, the *tet36* and *erm35* genes, encoding resistance to tetracyclines and macrolides respectively, were detected more frequently with the HT-qPCR than their counterpart *tetM* and *ermB*. This suggests that *tet36* and *erm35* are consistently associated with swine manure and able to be detected with current high throughput methods. The *tet36* gene was first discovered in swine manure pits, and is yet unclear whether it is enriched or persists in the environment upon manure application (Whittle et al., 2003; Kang et al., 2018; He et al., 2019). Less is known about the *erm35* gene, except that it was detected in poultry manure with metagenomics (Błażejewska et al., 2022; Wang and Chai, 2022). The *erm35* gene may have potential as a swine indicator since it was detected so frequently with HT-qPCR in the current study. One major difference between the two sets of genes is their association with mobile genetic elements (MGEs) where *ermB* and *tetM* are highly associated with MGEs while *erm35* and *tet36* are not (Zhang et al., 2022). MGEs are associated with the mobility of ARGs, which may be a significant variable for the dissemination of the gene after manure application. The class-3 MGE *inti3* was present in half of the manure samples tested in the ARG survey, and this is significant as this gene has the potential for horizontal gene transfer (Martínez et al., 2015). We highlight these genes *tet36* and *erm35* as potential targets for swine manure borne resistance.

## Conclusions

Overall, this study justifies the continued use of macrolide and tetracycline resistant ARGs as broad indicators of swine manure-borne resistance due to their presence in diverse manure samples. The observation of the concentrations of these genes in manures helps us to interpret whether abundances of these genes in the environment are substantial. Additionally, results of this study also highlight variations of using different methods to detect genes and their variability across ARGs. Due to the observed variation of ARGs in diverse manures, future studies should aim to characterize not only antibiotic residues, but also physiochemical properties of the manure to analyze for specific correlations that can explain this variability. We also provide supporting evidence that company integrator decisions may impact ARG concentrations, and we recommend future multidisciplinary studies to determine which company decisions may cause these observed differences.

The development of AMR bioindicators of manure impact is greatly needed for standardizing studies and for use in routine environmental monitoring (He et al., 2020; Howe and Soupir, 2021). This study provides support that standardized monitoring is likely but requires further evidence in development methods in gene selection and gene quantification. An ideal swine manure associated bioindicator should be commonly found in swine manure at the time of manure application and also specific to swine manure and not detected in natural environments. Often, the selection of ARGs are based on previous detection of ARGs, and our results justify the selection of these genes on broad manure samples. However, we also suggest that other genes within the tetracycline and erythromycin resistant classes may complement these genes and be more suitable for high-throughput methods. For detection of AMR impact in complex environments, like manures, it is likely that a single ARG will not be sufficient and methods that can detect and quantify multiple genes simultaneously provide opportunity for increased sensitivity and specificity of detection for monitoring efforts.

## Data availability statement

The datasets presented in this study can be found in online repositories. The names of the repository/repositories and accession number(s) can be found in the article/**Supplementary Material**.

## Ethics statement

Ethical review and approval was not required for the animal study because it is not needed in accordance with the local legislation and institutional requirements. This work was conducted in collaboration with local swine growers who made all animal decisions regarding health and well-being and allowed the collection of manure at their site. Written informed consent was obtained from the owners for the participation of their animals in this study.

## Author contributions

TN: Investigation, validation, data curation, formal analysis, writing – original draft, visualization. MS: Writing – review & editing. DA: Resources, writing – review & editing. MO: Investigation, writing - review & editing. AH: Conceptualization, methodology, resources, writing – review & editing, supervision, funding acquisition. All authors contributed to the article and approved the submitted version.

## References

[B1] Allen H. K. Donato J. Wang H. H. Cloud-Hansen K. A. Davies J. Handelsman J. (2010). Call of the wild: antibiotic resistance genes in natural environments. Nat. Rev. Microbiol. 8, 251–259. doi: 10.1038/nrmicro2312 20190823

[B2] Alt L. M. Iverson A. N. Soupir M. L. Moorman T. B. Howe A. (2021). Antibiotic resistance gene dissipation in soil microcosms amended with antibiotics and swine manure. J. Environ. Qual. 50, 911–922. doi: 10.1002/jeq2.20240 33982299

[B3] Bacanlı M. Başaran N. (2019). Importance of antibiotic residues in animal food. Food Chem. Toxicol. 125, 462–466. doi: 10.1016/j.fct.2019.01.033 30710599

[B4] Bates D. Mächler M. Bolker B. Walker S. (2015). Fitting linear mixed-effects models Usinglme4. J. Stat. Software 67, 1–48. doi: 10.18637/jss.v067.i01

[B5] Berendonk T. U. Manaia C. M. Merlin C. Fatta-Kassinos D. Cytryn E. Walsh F.. (2015). Tackling antibiotic resistance: the environmental framework. Nat. Rev. Microbiol. 13, 310–317. doi: 10.1038/nrmicro3439 25817583

[B6] Błażejewska A. Zalewska M. Grudniak A. Popowska M. (2022). A comprehensive study of the microbiome, resistome, and physical and chemical characteristics of chicken waste from intensive farms. Biomolecules 12, 1132. doi: 10.3390/biom12081132 36009027 PMC9406075

[B7] Center for Veterinary Medicine (2020) FDA 2019 report on antimicrobial sales for food-producing animals. Available at: https://www.fda.gov/animal-veterinary/cvm-updates/fda-releases-annual-summary-report-antimicrobials-sold-or-distributed-2019-use-food-producing (Accessed September 13, 2021).

[B8] Checcucci A. Trevisi P. Luise D. Modesto M. Blasioli S. Braschi I.. (2020). Exploring the animal waste resistome: The spread of antimicrobial resistance genes through the use of livestock manure. Front. Microbiol. 11, 1416. doi: 10.3389/fmicb.2020.01416 32793126 PMC7387501

[B9] Chee-Sanford J. C. Mackie R. I. Koike S. Krapac I. G. Lin Y.-F. Yannarell A. C.. (2009). Fate and transport of antibiotic residues and antibiotic resistance genes following land application of manure waste. J. Environ. Qual. 38, 1086–1108. doi: 10.2134/jeq2008.0128 19398507

[B10] Chen J. Yu Z. Michel F. C. Jr. Wittum T. Morrison M. (2007). Development and application of real-time PCR assays for quantification of erm genes conferring resistance to macrolides-lincosamides-streptogramin b in livestock manure and manure management systems. Appl. Environ. Microbiol. 73, 4407–4416. doi: 10.1128/AEM.02799-06 17496134 PMC1932836

[B11] Chen Q.-L. An X.-L. Li H. Zhu Y.-G. Su J.-Q. Cui L. (2017). Do manure-borne or indigenous soil microorganisms influence the spread of antibiotic resistance genes in manured soil? Soil Biol. Biochem. 114, 229–237. doi: 10.1016/j.soilbio.2017.07.022

[B12] Chen Z. Zhang W. Yang L. Stedtfeld R. D. Peng A. Gu C.. (2019). Antibiotic resistance genes and bacterial communities in cornfield and pasture soils receiving swine and dairy manures. Environ. pollut. 248, 947–957. doi: 10.1016/j.envpol.2019.02.093 30861417

[B13] Cheng D. Liu Y. Shehata E. Feng Y. Lin H. Xue J.. (2021). In-feed antibiotic use changed the behaviors of oxytetracycline, sulfamerazine, and ciprofloxacin and related antibiotic resistance genes during swine manure composting. J. Hazard. Mater. 402, 123710. doi: 10.1016/j.jhazmat.2020.123710 33254754

[B14] Cheng J. Tang X. Liu C. (2020). Occurrence and distribution of antibiotic resistance genes in various rural environmental media. Environ. Sci. pollut. Res. Int. 27, 29191–29203. doi: 10.1007/s11356-020-09287-x 32436087

[B15] Congilosi J. L. Wallace J. S. Neher T. P. Howe A. Soupir M. L. Aga D. S. (2022). Co-Occurrence of antimicrobials and metals as potential drivers of antimicrobial resistance in swine farms. Front. Environ. Sci. Eng. China 10. doi: 10.3389/fenvs.2022.1018739

[B16] Cooper O. (2018) How integration works on one US pig farm. farmers weekly. Available at: https://www.fwi.co.uk/livestock/pigs/how-integration-work-on-one-us-pig-farm (Accessed September 20, 2022).

[B17] Dewey C. E. Cox B. D. Straw B. E. Bush E. J. Hurd S. (1999). Use of antimicrobials in swine feeds in the united states. J. Swine Health Production 7, 19–25.

[B18] Dunlop R. H. McEwen S. A. Meek A. H. Friendship R. A. Clarke R. C. Black W. D. (1998). Antimicrobial drug use and related management practices among Ontario swine producers. Can. Vet. J. 39, 87–96. 10051955 PMC1539899

[B19] Elmund G. K. Morrison S. M. Grant D. W. Nevins S. M. (1971). Role of excreted chlortetracycline in modifying the decomposition process in feedlot waste. Bull. Environ. Contam. Toxicol. 6, 129–132. doi: 10.1007/BF01540093 5153752

[B20] Fang H. Han L. Zhang H. Long Z. Cai L. Yu Y. (2018). Dissemination of antibiotic resistance genes and human pathogenic bacteria from a pig feedlot to the surrounding stream and agricultural soils. J. Hazard. Mater. 357, 53–62. doi: 10.1016/j.jhazmat.2018.05.066 29860105

[B21] Fernanda P. A. Liu S. Yuan T. Ramalingam B. Lu J. Sekar R. (2022). Diversity and abundance of antibiotic resistance genes and their relationship with nutrients and land use of the inflow rivers of taihu lake. Front. Microbiol. 13, 1009297. doi: 10.3389/fmicb.2022.1009297 36267172 PMC9577174

[B22] Flater J. S. Alt L. M. Soupir M. Moorman T. B. Howe A. (2022). Prairie strips’ effect on transport of antimicrobial resistance indicators in poultry litter. J. Environ. Qual. 51, 260–271. doi: 10.1002/jeq2.20333 35112354

[B23] Funes-Huacca M. E. Opel K. Thompson R. McCord B. R. (2011). A comparison of the effects of PCR inhibition in quantitative PCR and forensic STR analysis. Electrophoresis 32, 1084–1089. doi: 10.1002/elps.201000584 21462225

[B24] Ghanbari M. Klose V. Crispie F. Cotter P. D. (2019). The dynamics of the antibiotic resistome in the feces of freshly weaned pigs following therapeutic administration of oxytetracycline. Sci. Rep. 9, 4062. doi: 10.1038/s41598-019-40496-8 30858509 PMC6411716

[B25] Gullberg E. Cao S. Berg O. G. Ilbäck C. Sandegren L. Hughes D.. (2011). Selection of resistant bacteria at very low antibiotic concentrations. PloS Pathog. 7, e1002158. doi: 10.1371/journal.ppat.1002158 21811410 PMC3141051

[B26] Hall M. C. Mware N. A. Gilley J. E. Bartelt-Hunt S. L. Snow D. D. Schmidt A. M.. (2020). Influence of setback distance on antibiotics and antibiotic resistance genes in runoff and soil following the land application of swine manure slurry. Environ. Sci. Technol. 54, 4800–4809. doi: 10.1021/acs.est.9b04834 32207931

[B27] Han X.-M. Hu H.-W. Chen Q.-L. Yang L.-Y. Li H.-L. Zhu Y.-G.. (2018). Antibiotic resistance genes and associated bacterial communities in agricultural soils amended with different sources of animal manures. Soil Biol. Biochem. 126, 91–102. doi: 10.1016/j.soilbio.2018.08.018

[B28] Hayes J. F. (2022). Fighting back against antimicrobial resistance with comprehensive policy and education: A narrative review. Antibiotics (Basel) 11, 644. doi: 10.3390/antibiotics11050644 35625288 PMC9137785

[B29] He L.-Y. He L.-K. Liu Y.-S. Zhang M. Zhao J.-L. Zhang Q.-Q.. (2019). Microbial diversity and antibiotic resistome in swine farm environments. Sci. Total Environ. 685, 197–207. doi: 10.1016/j.scitotenv.2019.05.369 31174117

[B30] He Y. Yuan Q. Mathieu J. Stadler L. Senehi N. Sun R.. (2020). Antibiotic resistance genes from livestock waste: occurrence, dissemination, and treatment. NPJ Clean Water 3, 4. doi: 10.1038/s41545-020-0051-0

[B31] Hölzel C. S. Müller C. Harms K. S. Mikolajewski S. Schäfer S. Schwaiger K.. (2012). Heavy metals in liquid pig manure in light of bacterial antimicrobial resistance. Environ. Res. 113, 21–27. doi: 10.1016/j.envres.2012.01.002 22280821

[B32] Howe A. C. Soupir M. L. (2021). Antimicrobial resistance in integrated agroecosystems: State of the science and future opportunities. J. Environ. Qual. 50, 1255–1265. doi: 10.1002/jeq2.20289 34528726

[B33] Hughes D. Andersson D. I. (2017). Environmental and genetic modulation of the phenotypic expression of antibiotic resistance. FEMS Microbiol. Rev. 41, 374–391. doi: 10.1093/femsre/fux004 28333270 PMC5435765

[B34] IADNR (2022) Confinements. iowa.gov. Available at: https://www.iowadnr.gov/Environmental-Protection/Animal-Feeding-Operations/Confinements (Accessed July 13, 2022).

[B35] IPPA (2012) Iowa Pork facts. Iowa pork. Available at: https://www.iowapork.org/news-from-the-iowa-pork-producers-association/iowa-pork-facts (Accessed September 29, 2022).

[B36] Joy S. R. Bartelt-Hunt S. L. Snow D. D. Gilley J. E. Woodbury B. L. Parker D. B.. (2013). Fate and transport of antimicrobials and antimicrobial resistance genes in soil and runoff following land application of swine manure slurry. Environ. Sci. Technol. 47, 12081–12088. doi: 10.1021/es4026358 24044357

[B37] Joy S. R. Li X. Snow D. D. Gilley J. E. Woodbury B. Bartelt-Hunt S. L. (2014). Fate of antimicrobials and antimicrobial resistance genes in simulated swine manure storage. Sci. Total Environ. 481, 69–74. doi: 10.1016/j.scitotenv.2014.02.027 24583946

[B38] Kang Y. Li Q. Yin Z. Shen M. Zhao H. Bai Y.. (2018). High diversity and abundance of cultivable tetracycline-resistant bacteria in soil following pig manure application. Sci. Rep. 8, 1489. doi: 10.1038/s41598-018-20050-8 29367695 PMC5784163

[B39] Kasuga I. Nagasawa K. Suzuki M. Kurisu F. Furumai H. (2022). High-throughput screening of antimicrobial resistance genes and their association with class 1 integrons in urban rivers in japan. Front. Environ. Sci. Eng. China 10. doi: 10.3389/fenvs.2022.825372

[B40] Keenum I. Liguori K. Calarco J. Davis B. C. Milligan E. Harwood V. J.. (2022). A framework for standardized qPCR-targets and protocols for quantifying antibiotic resistance in surface water, recycled water and wastewater. Crit. Rev. Environ. Sci. Technol. 52, 1–25. doi: 10.1080/10643389.2021.2024739

[B41] Klein E. Y. Van Boeckel T. P. Martinez E. M. Pant S. Gandra S. Levin S. A.. (2018). Global increase and geographic convergence in antibiotic consumption between 2000 and 2015. Proc. Natl. Acad. Sci. U. S. A. 115, E3463–E3470. doi: 10.1073/pnas.1717295115 29581252 PMC5899442

[B42] Lenth R. V. (2021) Emmeans: Estimated marginal means, aka least-squares means. Available at: https://CRAN.R-project.org/package=emmeans (Accessed January 15, 2022).

[B43] Li N. Zhu C. Liu C. Zhang X. Ding J. Zandi P.. (2019). The persistence of antimicrobial resistance and related environmental factors in abandoned and working swine feedlots. Environ. pollut. 255, 113116. doi: 10.1016/j.envpol.2019.113116 31622957

[B44] Lima T. Domingues S. Da Silva G. J. (2020). Manure as a potential hotspot for antibiotic resistance dissemination by horizontal gene transfer events. Vet. Sci. China 7, 110. doi: 10.3390/vetsci7030110 PMC7558842 32823495

[B45] Liu Z. Klümper U. Shi L. Ye L. Li M. (2019). From pig breeding environment to subsequently produced pork: Comparative analysis of antibiotic resistance genes and bacterial community composition. Front. Microbiol. 10, 43. doi: 10.3389/fmicb.2019.00043 30761096 PMC6361818

[B46] Looft T. Johnson T. A. Allen H. K. Bayles D. O. Alt D. P. Stedtfeld R. D.. (2012). In-feed antibiotic effects on the swine intestinal microbiome. Proc. Natl. Acad. Sci. U. S. A. 109, 1691–1696. doi: 10.1073/pnas.1120238109 22307632 PMC3277147

[B47] Lopatto E. Choi J. Colina A. Ma L. Howe A. Hinsa-Leasure S. (2019). Characterizing the soil microbiome and quantifying antibiotic resistance gene dynamics in agricultural soil following swine CAFO manure application. PloS One 14, e0220770. doi: 10.1371/journal.pone.0220770 31425534 PMC6699696

[B48] Lu X.-M. Li W.-F. Li C.-B. (2017). Characterization and quantification of antibiotic resistance genes in manure of piglets and adult pigs fed on different diets. Environ. pollut. 229, 102–110. doi: 10.1016/j.envpol.2017.05.080 28582673

[B49] Lüdecke D. Ben-Shachar M. Patil I. Waggoner P. Makowski D. (2021). Performance: An r package for assessment, comparison and testing of statistical models. J. Open Source Software 6, 3139. doi: 10.21105/joss.03139

[B50] Luo L. Feng J. Xue R. Ma J. Lou L. He J.. (2021). The insufficient extraction of DNA from swine manures may underestimate the abundance of antibiotic resistance genes as well as ignore their potential hosts. J. Environ. Manage. 278, 111587. doi: 10.1016/j.jenvman.2020.111587 33160229

[B51] Mackie R. I. Koike S. Krapac I. Chee-Sanford J. Maxwell S. Aminov R. I. (2006). Tetracycline residues and tetracycline resistance genes in groundwater impacted by swine production facilities. Anim. Biotechnol. 17, 157–176. doi: 10.1080/10495390600956953 17127527

[B52] Marti R. Tien Y.-C. Murray R. Scott A. Sabourin L. Topp E. (2014). Safely coupling livestock and crop production systems: How rapidly do antibiotic resistance genes dissipate in soil following a commercial application of swine or dairy manure? Appl. Environ. Microbiol. 80, 3258–3265. doi: 10.1128/aem.00231-14 24632259 PMC4018915

[B53] Martínez J. L. (2012). Natural antibiotic resistance and contamination by antibiotic resistance determinants: the two ages in the evolution of resistance to antimicrobials. Front. Microbiol. 3, 1. doi: 10.3389/fmicb.2012.00001 22275914 PMC3257838

[B54] Martínez J. L. Coque T. M. Baquero F. (2015). What is a resistance gene? ranking risk in resistomes. Nat. Rev. Microbiol. 13, 116–123. doi: 10.1038/nrmicro3399 25534811

[B55] McBride W. D. Key N. (2003). Economic and structural relationships in U.S. hog production (USDA-ERS Agricultural Economic Report No. 818). doi: 10.2139/ssrn.758464

[B56] Meyers M. A. Durso L. M. Gilley J. E. Waldrip H. M. Castleberry L. Millmier-Schmidt A. (2020). Antibiotic resistance gene profile changes in cropland soil after manure application and rainfall. J. Environ. Qual. 49, 754–761. doi: 10.1002/jeq2.20060 33016404

[B57] Mitchell J. O’Neill A. J. King R. (2022). Creating a framework to align antimicrobial resistance (AMR) research with the global guidance: a viewpoint. J. Antimicrob. Chemother. 77, dkac205. doi: 10.1093/jac/dkac205 35748621

[B58] Mu Q. Li J. Sun Y. Mao D. Wang Q. Luo Y. (2015). Occurrence of sulfonamide-, tetracycline-, plasmid-mediated quinolone- and macrolide-resistance genes in livestock feedlots in northern China. Environ. Sci. pollut. Res. Int. 22, 6932–6940. doi: 10.1007/s11356-014-3905-5 25475616

[B59] Muurinen J. Richert J. Wickware C. L. Richert B. Johnson T. A. (2021). Swine growth promotion with antibiotics or alternatives can increase antibiotic resistance gene mobility potential. Sci. Rep. 11, 5485. doi: 10.1038/s41598-021-84759-9 33750827 PMC7970892

[B60] Mware N. A. Hall M. C. Rajendran S. Gilley J. E. Schmidt A. M. Bartelt-Hunt S. L.. (2022). Resistome and mobilome in surface runoff from manured soil as affected by setback distance. J. Hazard. Mater. 429, 128278. doi: 10.1016/j.jhazmat.2022.128278 35065306

[B61] Neher T. P. Ma L. Moorman T. B. Howe A. Soupir M. L. (2020). Seasonal variations in export of antibiotic resistance genes and bacteria in runoff from an agricultural watershed in Iowa. Sci. Total Environ. 738, 140224. doi: 10.1016/j.scitotenv.2020.140224 32806354

[B62] OECD and Food and Agriculture Organization of the United Nations (2021). OECD-FAO agricultural outlook 2021-2030 (OECD Publishing, Paris: OECD).

[B63] Park S. Rana A. Sung W. Munir M. (2021). Competitiveness of quantitative polymerase chain reaction (qPCR) and droplet digital polymerase chain reaction (ddPCR) technologies, with a particular focus on detection of antibiotic resistance genes (ARGs). Appl. Microbiol. 1, 426–444. doi: 10.3390/applmicrobiol1030028

[B64] Pärnänen K. Karkman A. Hultman J. Lyra C. Bengtsson-Palme J. Larsson D. G. J.. (2018). Maternal gut and breast milk microbiota affect infant gut antibiotic resistome and mobile genetic elements. Nat. Commun. 9, 3891. doi: 10.1038/s41467-018-06393-w 30250208 PMC6155145

[B65] Peng S. Feng Y. Wang Y. Guo X. Chu H. Lin X. (2017). Prevalence of antibiotic resistance genes in soils after continually applied with different manure for 30 years. J. Hazard. Mater. 340, 16–25. doi: 10.1016/j.jhazmat.2017.06.059 28711829

[B66] Petrin S. Patuzzi I. Di Cesare A. Tiengo A. Sette G. Biancotto G.. (2019). Evaluation and quantification of antimicrobial residues and antimicrobial resistance genes in two Italian swine farms. Environ. pollut. 255, 113183. doi: 10.1016/j.envpol.2019.113183 31541814

[B67] Radu E. Woegerbauer M. Rab G. Oismüller M. Strauss P. Hufnagl P.. (2021). Resilience of agricultural soils to antibiotic resistance genes introduced by agricultural management practices. Sci. Total Environ. 756, 143699. doi: 10.1016/j.scitotenv.2020.143699 33307498

[B68] Reimer J. J. (2006). Vertical integration in the pork industry. Am. J. Agric. Econ. 88, 234–248. doi: 10.1111/j.1467-8276.2006.00850.x

[B69] Samanta P. Horn H. Saravia F. (2022). Removal of diverse and abundant ARGs by MF-NF process from pig manure and digestate. Membranes 12, 661. doi: 10.3390/membranes12070661 35877864 PMC9317629

[B70] Sandberg K. D. Ishii S. LaPara T. M. (2018). A microfluidic quantitative polymerase chain reaction method for the simultaneous analysis of dozens of antibiotic resistance and heavy metal resistance genes. Environ. Sci. Technol. Lett. 5, 20–25. doi: 10.1021/acs.estlett.7b00552

[B71] Shui J. Tuo H. Liu J. Zhang X. Feng J. Feng Y.. (2022). Insights into the fates of plasmids and antimicrobial resistance genes during swine manure treatment and related factors based on plasmidome and metagenome analyses. Environ. Sci. pollut. Res. Int 29, 69037–69047. doi: 10.1007/s11356-022-20574-7 35562609

[B72] Sidstedt M. Rådström P. Hedman J. (2020). PCR inhibition in qPCR, dPCR and MPS-mechanisms and solutions. Anal. Bioanal. Chem. 412, 2009–2023. doi: 10.1007/s00216-020-02490-2 32052066 PMC7072044

[B73] Stedtfeld R. D. Guo X. Stedtfeld T. M. Sheng H. Williams M. R. Hauschild K.. (2018). Primer set 2.0 for highly parallel qPCR array targeting antibiotic resistance genes and mobile genetic elements. FEMS Microbiol. Ecol. 94, fiy130. doi: 10.1093/femsec/fiy130 30052926 PMC7250373

[B74] Tsoulouhas T. Vukina T. (1999). Integrator contracts with many agents and bankruptcy. Am. J. Agric. Econ. 81, 61–74. doi: 10.2307/1244450

[B75] Van Goethem M. W. Pierneef R. Bezuidt O. K. I. Van De Peer Y. Cowan D. A. Makhalanyane T. P. (2018). A reservoir of ‘historical’ antibiotic resistance genes in remote pristine Antarctic soils. Microbiome 6, 40. doi: 10.1186/s40168-018-0424-5 29471872 PMC5824556

[B76] Wang L. Chai B. (2022). Fate of antibiotic resistance genes and changes in bacterial community with increasing breeding scale of layer manure. Front. Microbiol. 13, 857046. doi: 10.3389/fmicb.2022.857046 35356511 PMC8959713

[B77] Wang F. Han W. Chen S. Dong W. Qiao M. Hu C.. (2020). Fifteen-year application of manure and chemical fertilizers differently impacts soil ARGs and microbial community structure. Front. Microbiol. 11, 62. doi: 10.3389/fmicb.2020.00062 32117108 PMC7015874

[B78] Waseem H. Saleem ur Rehman H. Ali J. Iqbal M. J. Ali M. I. (2020). “Global trends in ARGs measured by HT-qPCR platforms,” in Antibiotics and antimicrobial resistance genes in the environment. Ed. Hashmi M. Z. (Elsevier), 206–222.

[B79] Wellington E. M. H. Boxall A. B. Cross P. Feil E. J. Gaze W. H. Hawkey P. M.. (2013). The role of the natural environment in the emergence of antibiotic resistance in gram-negative bacteria. Lancet Infect. Dis. 13, 155–165. doi: 10.1016/S1473-3099(12)70317-1 23347633

[B80] Wen X. Mi J. Wang Y. Ma B. Zou Y. Liao X.. (2019). Occurrence and contamination profiles of antibiotic resistance genes from swine manure to receiving environments in guangdong province southern China. Ecotoxicol. Environ. Saf. 173, 96–102. doi: 10.1016/j.ecoenv.2019.02.023 30769208

[B81] Whitehead T. R. Cotta M. A. (2013). Stored swine manure and swine faeces as reservoirs of antibiotic resistance genes. Lett. Appl. Microbiol. 56, 264–267. doi: 10.1111/lam.12043 23297734

[B82] Whittle G. Whitehead T. R. Hamburger N. Shoemaker N. B. Cotta M. A. Salyers A. A. (2003). Identification of a new ribosomal protection type of tetracycline resistance gene, tet(36), from swine manure pits. Appl. Environ. Microbiol. 69, 4151–4158. doi: 10.1128/AEM.69.7.4151-4158.2003 12839793 PMC165177

[B83] Xue J. Wu J. Hu Y. Sha C. Yao S. Li P.. (2021). Occurrence of heavy metals, antibiotics, and antibiotic resistance genes in different kinds of land-applied manure in China. Environ. Sci. pollut. Res. Int. 28, 40011–40021. doi: 10.1007/s11356-021-13307-9 33768462

[B84] Yang F. Han B. Gu Y. Zhang K. (2020). Swine liquid manure: a hotspot of mobile genetic elements and antibiotic resistance genes. Sci. Rep. 10, 15037. doi: 10.1038/s41598-020-72149-6 32929149 PMC7490410

[B85] Zalewska M. Błażejewska A. Czapko A. Popowska M. (2021). Antibiotics and antibiotic resistance genes in animal manure - consequences of its application in agriculture. Front. Microbiol. 12, 610656. doi: 10.3389/fmicb.2021.610656 33854486 PMC8039466

[B86] Zhang B. Tian H. Lu C. Dangal S. R. S. Yang J. Pan S. (2017). Global manure nitrogen production and application in cropland during 1860–2014: a 5 arcmin gridded global dataset for earth system modeling. Earth System Sci. Data 9, 667. doi: 10.5194/essd-9-667-2017

[B87] Zhang K. Xin R. Zhao Z. Li W. Wang Y. Wang Q.. (2021). Mobile genetic elements are the major driver of high antibiotic resistance genes abundance in the upper reaches of huaihe river basin. J. Hazard. Mater. 401, 123271. doi: 10.1016/j.jhazmat.2020.123271 32629348

[B88] Zhang Z. Zhang Q. Wang T. Xu N. Lu T. Hong W.. (2022). Assessment of global health risk of antibiotic resistance genes. Nat. Commun. 13, 1553. doi: 10.1038/s41467-022-29283-8 35322038 PMC8943045

[B89] Zhao X. Wang J. Zhu L. Wang J. (2019). Field-based evidence for enrichment of antibiotic resistance genes and mobile genetic elements in manure-amended vegetable soils. Sci. Total Environ. 654, 906–913. doi: 10.1016/j.scitotenv.2018.10.446 30453260

[B90] Zhou X. Wang J. Lu C. Liao Q. Gudda F. O. Ling W. (2020). Antibiotics in animal manure and manure-based fertilizers: Occurrence and ecological risk assessment. Chemosphere 255, 127006. doi: 10.1016/j.chemosphere.2020.127006 32417517

[B91] Zhu Y.-G. Johnson T. A. Su J.-Q. Qiao M. Guo G.-X. Stedtfeld R. D.. (2013). Diverse and abundant antibiotic resistance genes in Chinese swine farms. Proc. Natl. Acad. Sci. U. S. A. 110, 3435–3440. doi: 10.1073/pnas.1222743110 23401528 PMC3587239

