# Supplementary material for: Comparison of antibiotic resistance genes in swine manure storage pits of Iowa, USA
Source: Front Antibiot. 2023 Mar 23;2:1116785. doi: 10.3389/frabi.2023.1116785 (PMC11732143; doi:10.3389/frabi.2023.1116785)
Supplement: Supplementary file 1 [file DataSheet_1.docx]

Supplementary Material

Comparison of antibiotic resistance genes in swine manures of Iowa, USA

**Timothy P. Neher^*^, Michelle L. Soupir, Daniel S. Andersen, Maggie L. O’Neill, Adina Howe**

*** Correspondence:** Corresponding Author: [tpneher@iastate.edu](mailto:tpneher@iastate.edu)

**Data Availability:** The datasets generated and analyzed for this study can be found on GitHub at https://github.com/tpneher/manure-amr-indicators.

# Supplementary Tables

**Supplementary Table 1.** Table of primers (Stedtfeld et al. 2018).

| **Gene** | **Forward Primer Sequence (5'-3')** | **Reverse Primer Sequence (5'-3')** |
| --- | --- | --- |
| 16S rRNA | ATG GTT GTC GTC AGC T | ACG GGC GGT GTG TAC |
| aadD | CCG ACA ACA TTT CTA CCA TCC TT | ACC GAA GCG CTC GTC GTA TA |
| aadA2 | ACG GCT CCG CAG TGG AT | GGC CAC AGT AAC CAA CAA ATC A |
| blaPSE | TTG TGA CCT ATT CCC CTG TAA TAG AA | TGC GAA GCA CGC ATC ATC |
| blaOXA10 | CGA CCG AGT ATG TAC CTG CTT C | TCA AGT CCA ATA CGA CGA GCT A |
| cmlA1 | TAG GAA GCA TCG GAA CGT TGA T | CAG ACC GAG CAC GAC TGT TG |
| cmlA5 | GCG CTC TTC GAG GAT TCG | CCG CCC AAG CAG AAG TAG AC |
| erm(35) | CCT TCA GTC AGA ACC GGC AA | GCT GAT TTG ACA GTT GGT GGT G |
| erm(36) | GGC GGA CCG ACT TGC AT | TCT GCG TTG ACG ACG GTT AC |
| ermB | GAA CAC TAG GGT TGT TCT TGC A | CTG GAA CAT CTG TGG TAT GGC |
| ermC | TTT GAA ATC GGC TCA GGA AAA | ATG GTC TAT TTC AAT GGC AGT TAC G |
| ermF | TCT GAT GCC CGA AAT GTT CAA G | TGA AGG ACA ATT GAA CCT CCC A |
| ermQ | TGA AAG CCA TGC GTC TGA C | TTC AGC TGG CAG CTT AAG C |
| ermT | GTT CAC TAG CAC TAT TTT TAA TGA CAG AAG T | GAA GGG TGT CTT TTT AAT ACA ATT AAC GA |
| floR | AAC CCG CCC TCT GGA TCA | GCC GTC GAG AAG AAG ACG AA |
| intl2 | TGC TTT TCC CAC CCT TAC C | GAC GGC TAC CCT CTG TTA TCT C |
| intl3 | CAG GTG CTG GGC ATG GA | CCT GGG CAG CAT CAC CA |
| intl1F165-clinical | CGA ACG AGT GGC GGA GGG TG | TAC CCG AGA GCT TGG CAC CCA |
| intl1-a-marko | CGA AGT CGA GGC ATT TCT GTC | GCC TTC CAG AAA ACC GAG GA |
| lnuA | TGA CGC TCA ACA CAC TCA AAA A | TTC ATG CTT AAG TTC CAT ACG TGA A |
| lnuC | GGG TGT AGA TGC TCT TCT TGG A | CTT TAC CCG AAA GAG TTT CTA CCG |
| sul1 NEW | GCC GAT GAG ATC AGA CGT ATT G | CGC ATA GCG CTG GGT TTC |
| sul2 | TCA TCT GCC AAA CTC GTC GTT A | GTC AAA GAA CGC CGC AAT GT |
| str | AAT GAG TTT TGG AGT GTC TCA ACG TA | AAT CAA AAC CCC TAT TAA AGC CAA T |
| tet(36) | AGA ATA CTC AGC AGA GGT CAG TTC CT | TGG TAG GTC GAT AAC CCG AAA AT |
| tetA | CTC ACC AGC CTG ACC TCG AT | CAC GTT GTT ATA GAA GCC GCA TAG |
| tetL | ATG GTT GTA GTT GCG CGC TAT AT | ATC GCT GGA CCG ACT CCT T |
| tetM | GGA GCG ATT ACA GAA TTA GGA AGC | TCC ATA TGT CCT GGC GTG TC |
| tetO | CAA CAT TAA CGG AAA GTT TAT TGT ATA CCA | TTG ACG CTC CAA ATT CAT TGT ATC |
| tetT | CCA TAT AGA GGT TCC ACC AAA TCC | TGA CCC TAT TGG TAG TGG TTC TAT TG |
| tetW | ATG AAC ATT CCC ACC GTT ATC TTT | ATA TCG GCG GAG AGC TTA TCC |
| tetX | AAA TTT GTT ACC GAC ACG GAA GTT | CAT AGC TGA AAA AAT CCA GGA CAG TT |

**Supplementary Table 2.** Conventional qPCR standard curve performance metrics and associated limits of quantification (LOQ in copies/µL DNA template).

| **Gene** | **Plate #** | **Slope** | **R^2** | **y-intercept** | **Efficiency** | **LOQ** |
| --- | --- | --- | --- | --- | --- | --- |
| **16S** | 1 | -3.75 | 0.997 | 38.69 | 84.7 | 31 |
|  | 2 | -3.73 | 0.995 | 38.83 | 85.3 | 38 |
|  | 3 | -3.70 | 0.995 | 38.70 | 86.3 | 38 |
|  | 4 | -3.74 | 0.991 | 38.85 | 85.0 | 53 |
|  | 5 | -3.70 | 0.992 | 38.84 | 86.2 | 43 |
|  | 6 | -3.78 | 0.984 | 39.02 | 83.8 | 58 |
|  | 7 | -3.81 | 0.996 | 38.93 | 83.1 | 34 |
| ***ermB*** | 1 | -3.84 | 0.999 | 41.29 | 82.2 | 37 |
|  | 2 | -3.74 | 0.997 | 40.52 | 84.9 | 48 |
|  | 3 | -3.65 | 0.996 | 40.31 | 87.9 | 37 |
|  | 4 | -3.73 | 0.998 | 40.77 | 85.3 | 45 |
|  | 5 | -3.73 | 0.998 | 40.52 | 85.3 | 40 |
|  | 6 | -3.40 | 0.994 | 38.00 | 96.7 | 112 |
|  | 7 | -3.31 | 0.996 | 37.44 | 100.6 | 74 |
| ***tetM*** | 1 | -3.55 | 1.000 | 37.59 | 91.1 | 19 |
|  | 2 | -3.51 | 0.999 | 37.17 | 92.6 | 20 |
|  | 3 | -3.63 | 0.999 | 38.08 | 88.4 | 15 |
|  | 4 | -3.46 | 0.999 | 36.93 | 94.4 | 19 |
|  | 5 | -3.58 | 0.998 | 38.01 | 90.1 | 25 |
|  | 6 | -3.71 | 0.999 | 38.93 | 85.9 | 21 |
|  | 7 | -3.79 | 0.999 | 39.39 | 83.7 | 18 |

**Supplementary Table 3.** HT-qPCR standard curve performance metrics and associated limits of quantification (LOQ in copies/µL DNA template).

|  | **Gene** | **Slope** | **R^2** | **y-intercept** | **Efficiency** | **LOQ** |
| --- | --- | --- | --- | --- | --- | --- |
| **Biomark Chip 1** | 16S rRNA | -3.76 | 0.992 | 25.78 | 84.5 | 30,015 |
|  | *ermB* | -3.47 | 0.991 | 25.09 | 94.1 | 4,209 |
|  | *ermF* | -3.34 | 0.989 | 24.26 | 99.5 | 3,343 |
|  | *sul2* | -3.53 | 0.982 | 25.93 | 92.1 | 4,582 |
|  | *tetM* | -3.45 | 0.998 | 24.42 | 95.0 | 1,612 |
|  | *tetW* | -3.39 | 0.995 | 24.86 | 97.2 | 1,493 |
| **Biomark Chip 2** | 16S rRNA | -3.92 | 0.968 | 27.01 | 80.0 | 49,284 |
|  | *ermB* | -3.55 | 0.991 | 25.76 | 91.2 | 2,881 |
|  | *ermF* | -3.30 | 0.979 | 24.34 | 100.9 | 2,701 |
|  | *sul2* | -3.29 | 0.962 | 25.46 | 101.4 | 7,791 |
|  | *tetM* | -3.51 | 0.990 | 24.98 | 92.8 | 1,637 |
|  | *tetW* | -3.42 | 0.992 | 25.07 | 96.0 | 1,269 |
| **Biomark Chip 3** | 16S rRNA | -3.70 | 0.985 | 26.06 | 86.4 | 28,582 |
|  | *ermB* | -3.51 | 0.985 | 25.86 | 92.7 | 3,539 |
|  | *ermF* | -3.23 | 0.954 | 24.31 | 104.2 | 2,551 |
|  | *sul2* | -3.56 | 0.940 | 27.62 | 91.1 | 7,649 |
|  | *tetM* | -3.46 | 0.989 | 24.99 | 94.5 | 2,149 |
|  | *tetW* | -3.56 | 0.992 | 26.19 | 90.8 | 564 |

**Supplementary Table 4.** Linear regression model performance.

| **Model Performance** | | | | | | | | |
| --- | --- | --- | --- | --- | --- | --- | --- | --- |
| **Response** | **Gene** | **Fixed Effects** | **AIC** | **BIC** | **R^2** | **R^2 (adjusted)** | **RMSE** | **Sigma** |
| Copies/g | ermB | Integrator, Production System | 114.9 | 124.22 | 0.266 | 0.216 | 0.721 | 0.753 |
| Copies/g | tetM | Integrator, Production System | 116.4 | 125.72 | 0.092 | 0.03 | 0.733 | 0.765 |
| Relative Abundance | ermB | Integrator, Production System | 70.75 | 80.104 | 0.316 | 0.269 | 0.456 | 0.476 |
| Relative Abundance | tetM | Integrator, Production System | 59.98 | 69.333 | 0.35 | -0.031 | 0.407 | 0.425 |

**Supplementary Table 5.** ANOVA of gene concentrations A) (log10 copies/g manure wet weight), B) (log10 relative abundance) across all 48 manure samples.

| **ARG** | **n** | **F ratio** | **P value** |
| --- | --- | --- | --- |
| A) |  |  |  |
| *ermB* | 48 | 7.573 | <0.0001 |
| *tetM* | 48 | 7.573 | <0.0001 |
| B) |  |  |  |
| *ermB*  *tetM* | 48  48 | 2.092  2.092 | 0.0064  0.0064 |

**Supplementary Table 6.** A) The ANOVA of estimated marginal means (log10 copies/g) for each effect when averaged over other fixed effects in each linear model. B) The calculated estimated marginal means (log10), standard errors (SE), lower confidence levels (LCL), and upper confidence levels (UCL) of integrator effects. C) Production systems estimated marginal means (log10).

| A) | | | | | *ermB* | | | | | | *tetM* | | | | |
| --- | --- | --- | --- | --- | --- | --- | --- | --- | --- | --- | --- | --- | --- | --- | --- |
|  | | | | | F ratio | | | P-value | | | F ratio | | | | P-value |
| Integrator | | | | | 13.293 | | | 0.0007 | | | 4.363 | | | | 0.0425 |
| Production System | | | | | 0.491 | | | 0.4872 | | | 0.014 | | | | 0.9074 |
| Integrator:Production System | | | | | 0.034 | | | 0.8546 | | | 1.213 | | | | 0.2767 |
| B) | Integrator | n Farms | | | Estimated Marginal Mean | SE | | | LCL | | UCL | |  |  |  |
| *ermB* | 1 | 24 | | | 5.81 | 0.169 | | | 5.47 | | 6.15 | |  |  |  |
|  | 2 | 24 | | | 6.68 | 0.169 | | | 6.34 | | 7.02 | |  |  |  |
| *tetM* | 1 | 24 | | | 6.08 | 0.172 | | | 5.74 | | 6.43 | |  |  |  |
|  | 2 | 24 | | | 6.59 | 0.172 | | | 6.24 | | 6.94 | |  |  |  |
| C) | Production System | | n Farms | Estimated Marginal Mean | | | SE | | | LCL | | UCL | |  |  |
| *ermB* | GF | | 14 | 6.33 | | | 0.201 | | | 5.92 | | 6.73 | |  |  |
|  | WF | | 34 | 6.16 | | | 0.129 | | | 5.90 | | 6.42 | |  |  |
| *tetM* | GF | | 14 | 6.32 | | | 0.205 | | | 5.91 | | 6.74 | |  |  |
|  | WF | | 34 | 6.35 | | | 0.131 | | | 6.09 | | 6.62 | |  |  |

**Supplementary Table 7.** A) The ANOVA of estimated marginal means (Log10 relative abundance) for each effect when averaged over other fixed effects in each linear model. B) The calculated estimated marginal means (log10), standard errors (SE), lower confidence levels (LCL), and upper confidence levels (UCL) of integrator effects. C) Production systems estimated marginal means (log10).

| A) | | | | | *ermB* | | | | | | *tetM* | | |
| --- | --- | --- | --- | --- | --- | --- | --- | --- | --- | --- | --- | --- | --- |
|  | | | | | F ratio | | | P-value | | | F ratio | | P-value |
| Integrator | | | | | 10.77 | | | 0.002 | | | 0.831 | | 0.367 |
| Production System | | | | | 0.441 | | | 0.510 | | | 0.680 | | 0.414 |
| Integrator:Production System | | | | | 1.881 | | | 0.177 | | | 0.011 | | 0.918 |
| B) | Integrator | n Farms | | | Estimated Marginal Mean | SE | | | LCL | | UCL |  |  |
| *ermB* | 1 | 24 | | | -1.128 | 0.107 | | | -1.343 | | -0.912 |  |  |
|  | 2 | 24 | | | -0.632 | 0.107 | | | -0.514 | | -0.416 |  |  |
| *tetM* | 1 | 24 | | | -0.853 | 0.096 | | | -1.045 | | -0.660 |  |  |
|  | 2 | 24 | | | -0.730 | 0.096 | | | -0.922 | | -0.537 |  |  |
| C) | Production System | | n Farms | Estimated Marginal Mean | | | SE | | | LCL | UCL |  |  |
| *ermB* | GF | | 14 | -0.830 | | | 0.127 | | | -1.090 | 0.573 |  |  |
|  | WF | | 34 | -0.930 | | | 0.0816 | | | -1.090 | -0.765 |  |  |
| *tetM* | GF | | 14 | -0.847 | | | 0.114 | | | -1.018 | -0.618 |  |  |
|  | WF | | 34 | -0.736 | | | 0.073 | | | -0.883 | -0.589 |  |  |

# Supplementary Figures


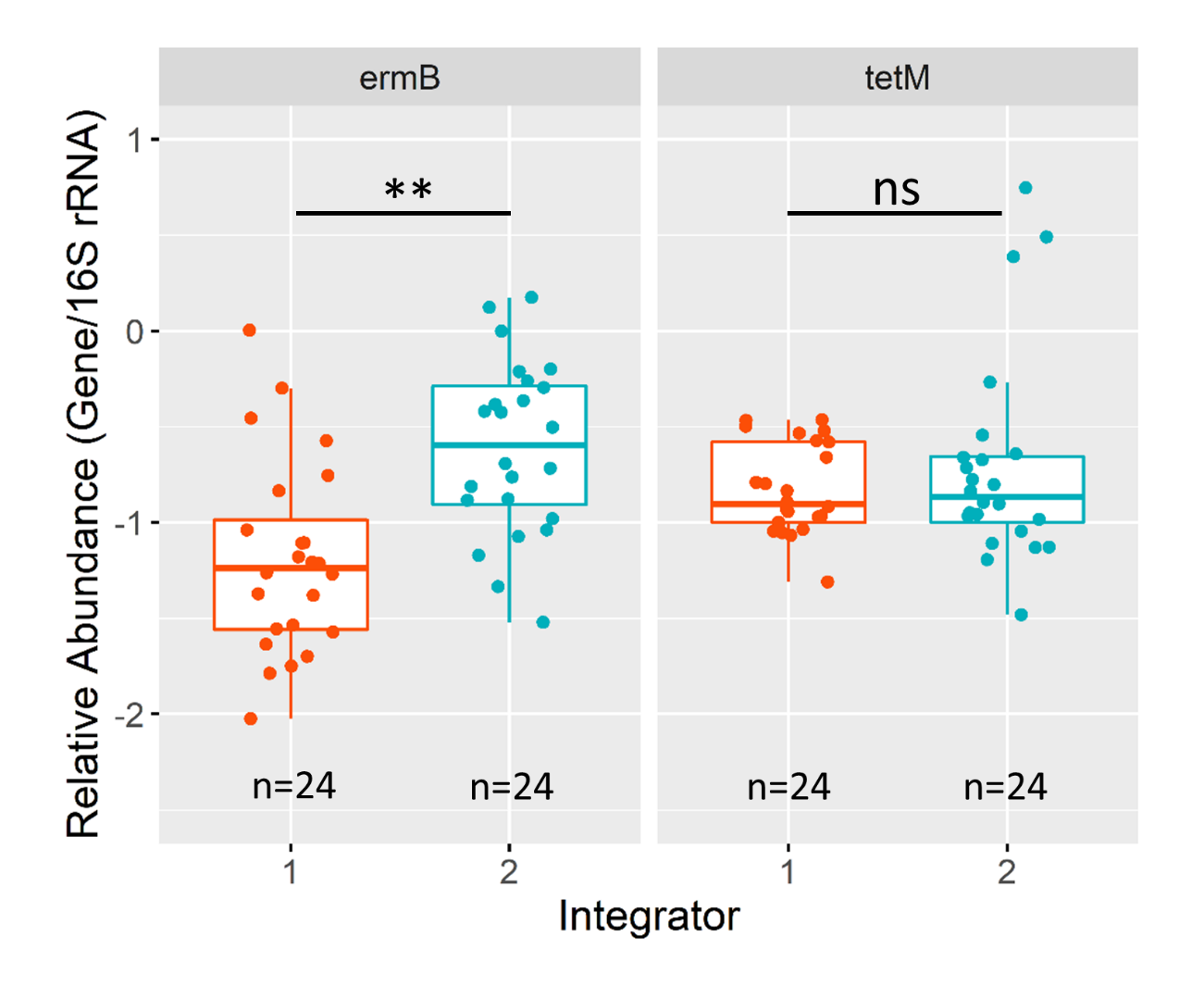


**Supplementary Figure 1.** Relative abundance of *ermB* and *tetM* to 16S rRNA gene grouped by company integrator. Asterisks above boxplots signify p-values (alpha = 0.05) based on results of the linear model (not significant [ns] p>0.05, * p<0.05, ** p<0.01, *** p<0.001, **** p<0.00001). Interquartile ranges are indicated by boxes and the upper 25% and lower 25% are indicated by whiskers. The number of farms (n) are labelled on the x-axis


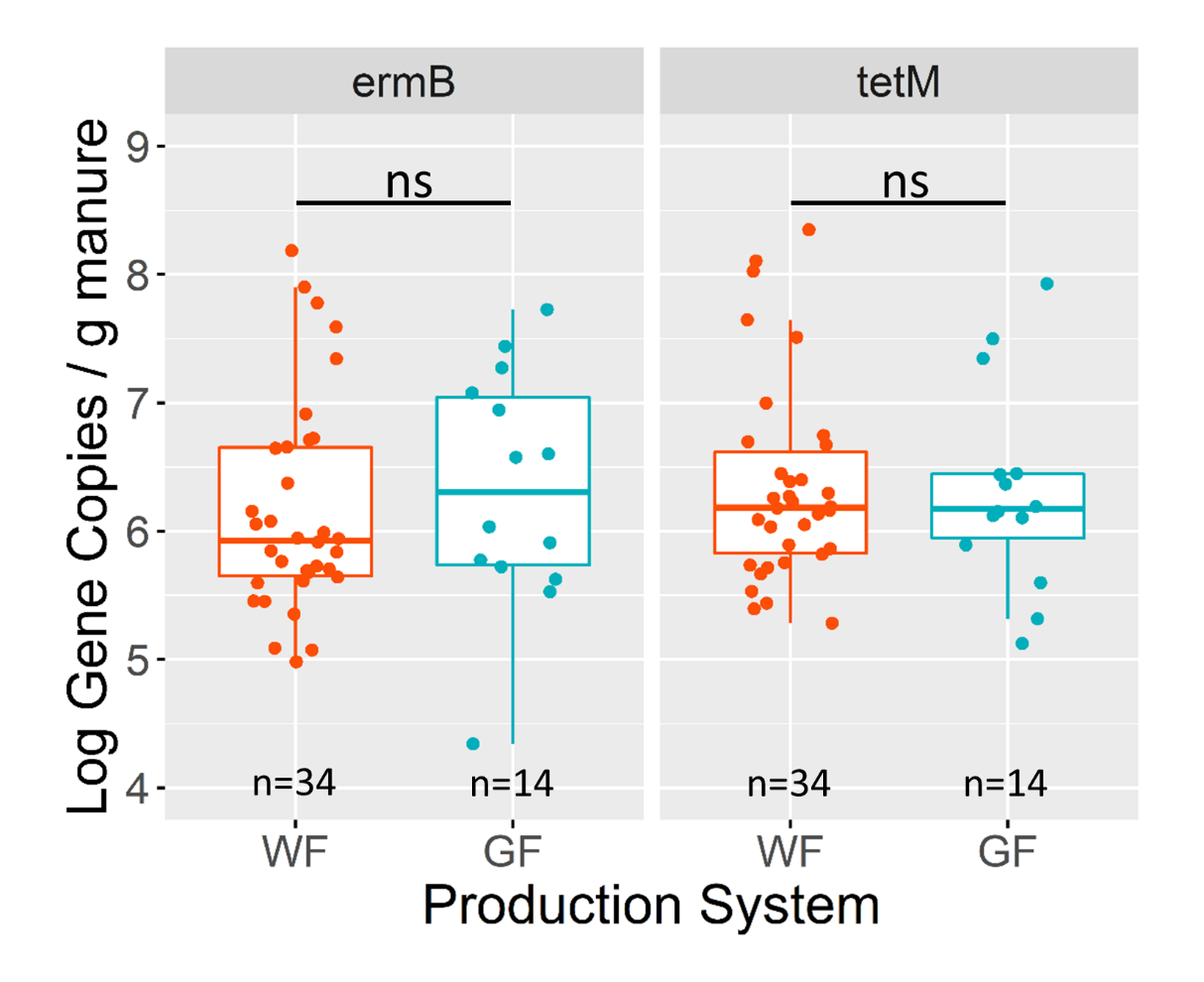


**Supplementary Figure 2.** Log10 Gene copies of *ermB* and *tetM* grouped by production system. Asterisks above boxplots signify p-values based on results of the linear model (not significant [ns] p>0.05, * p<0.05, ** p<0.01, *** p<0.001, **** p<0.00001). Interquartile ranges are indicated by boxes and the upper 25% and lower 25% are indicated by whiskers. The number of farms (n) are labelled on the x-axis. WF (wean-finish), GF (grow-finish)


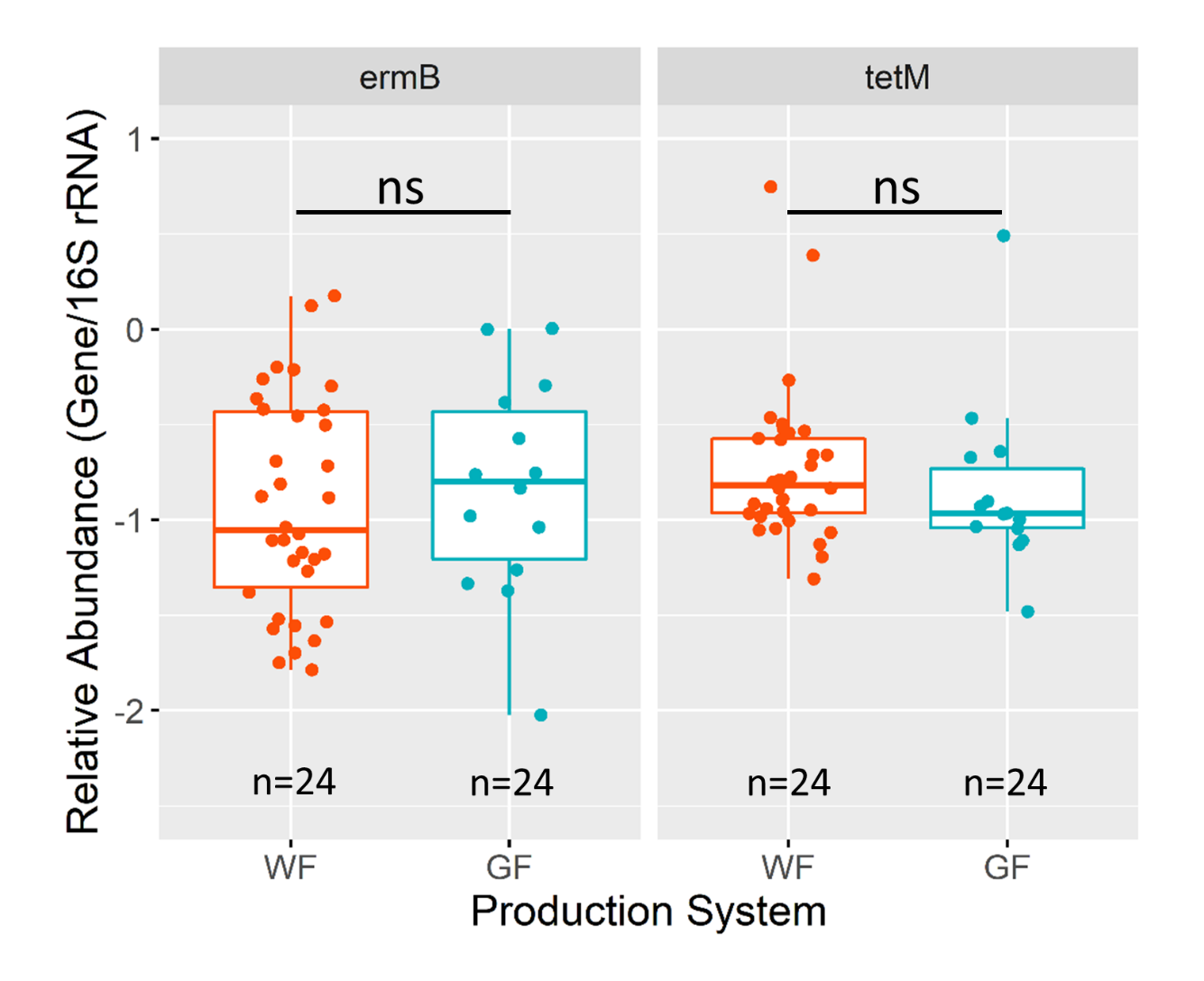


**Supplementary Figure 3.** Relative abundance of *ermB* and *tetM* to 16S rRNA gene grouped by production system. Asterisks above boxplots signify p-values based on results of the linear model (not significant [ns] p>0.05, * p<0.05, ** p<0.01, *** p<0.001, **** p<0.00001). Interquartile ranges are indicated by boxes and the upper 25% and lower 25% are indicated by whiskers. The number of farms (n) are labelled on the x-axis. WF (wean-finish), GF (grow-finish)
